# Transcription Factor Analysis of Rhodophytes Suggests Trihelix Transcription Factors Across the Florideophyceae

**DOI:** 10.3390/plants14203143

**Published:** 2025-10-12

**Authors:** Lachlan J. McKinnie, Scott F. Cummins, Sankar Subramanian, Min Zhao

**Affiliations:** 1 Centre for BioInnovation, University of the Sunshine Coast, Maroochydore, QLD 4558, Australia; lachlan.mckinnie@research.usc.edu.au (L.J.M.); scummins@usc.edu.au (S.F.C.); ssankara@usc.edu.au (S.S.); 2 School of Science, Technology and Engineering, University of the Sunshine Coast, Maroochydore, QLD 4558, Australia

**Keywords:** transcription factors, trihelix, *Asparagopsis taxiformis*, Rhodophyta, red algae

## Abstract

Transcription factors (TFs) are important gene transcription regulators involved in myriad functions such as development, metabolism, and stress response. TFs are found in all eukaryotes, with many families of TFs unique to plants and algae. Algae are of interest due to a wide range of novel metabolites, of which TFs play an important role in regulating their biosynthesis. In particular, the red algae (phylum Rhodophyta) are a source of several important metabolites that are a current focus of further research. However, to date, investigations of TF families in rhodophytes have been limited due to the relative lack of genomic resources available and the small number of in silico analyses of their TFs. In this study, we used genomic and transcriptomic data to identify rhodophyte TFs. We found that the general proportion of TFs in rhodophytes was overall consistent with previous research. However, for the first time in the rhodophyte class Florideophyceae, we report the presence of a putative TF within the trihelix TF (TTF) family, which are light-sensitive TFs associated with growth and stress response. In particular, we demonstrate evidence suggesting the presence of putative TTFs in three *Asparagopsis taxiformis* genomes, as well as in several other florideophyte assemblies. This was supported by analyses including Neighbour-Joining phylogeny, protein structure prediction, and motif analysis. In summary, this research reported the repertoire of TFs in rhodophyte algae across a much greater range than previously reported and identified putative TTFs in several algae from the class Florideophyceae. This opens an avenue for further research into the evolution of various TFs in early plants, as well as key regulatory factors in rhodophyte metabolism, though future research, such as functional characterisation, will be required to confirm these findings.

## 1. Introduction

Transcription factors (TFs) regulate the transcription of target genes through the activation or inactivation of upstream regulatory elements [1]. All protein-coding genes in eukaryotes require TFs, in conjunction with other regulatory elements, to initiate transcription [2]. TFs are some of the most heavily expanded gene families among plants, which have facilitated functional divergence and adaptive traits in plants, such as adaptation to terrestrial or otherwise hostile environments. This has led to increased subfunctionalisation, with the expansion of TFs largely due to high rates of gene duplication, as well as high rates of gene retention [3,4], with expansion due to mechanisms such as whole genome duplication and tandem gene duplication [3]. TFs are heavily conserved across organisms, even across different phyla. For example, many TFs found in land plants are also found in algal lineages such as red algae (Rhodophyta), green algae (Chlorophyta), and Haptophyta [5], including TFs with homologous functions between algae and land plants [6].

Numerous different TF families have been found in rhodophytes, of which the C2H2 family is typically the most abundant, often comprising approximately 20–40% of total TFs, barring the polyextremophilic class Cyanidiophyceae, where they typically only make up around 5–10% [5,7,8,9]. By comparison, the myeloblastosis (MYB) TF family is less abundant, but no less important, with regulatory impacts including effects on secondary metabolism, stress response, and signalling pathways [10], such as the R2R3-MYB factor, which regulates flavanol biosynthesis in *Epimedium sagittatum* [11]. The MYB family is also incredibly diverse, and there is a wide range of related factors categorised as MYB-related families, while more still have been described as evolutionary descendants of MYB genes, such as the trihelix family of TFs (TTFs), also known as the GT family, as they bind to the GT element on the DNA sequence [12]. TTFs are primarily found in plants and are composed of a helix–loop–helix–loop–helix structure [12,13], with the helices having high structural and sequence similarity to MYB factors [12]. Five families of TTFs (GT-1, GT-2, GTγ, SH4, and SIP1) have been described in *Arabidopsis* [14]. The GT-1 protein contains a single trihelix DNA-binding domain, whereas the GT-2 protein contains two similar trihelix domains [15]. By contrast, MYB TFs are classified depending on the number of different repeats (one to four). The MYB-related class of MYB TFs comprises proteins with a single or partial MYB repeat [16] and are associated with cellular morphogenesis and control of secondary metabolism [17,18].

TTFs are involved in the regulation of various genes in plants, including seed maturation [19], cytochrome P450 reductase, chalcone synthases, phytochrome A, plastocyanin, ATP synthase, and others [20]. TTFs possess a wide range of functions. They have been reported to act as light-specific regulators in a wide range of contexts, but also bind to non-light-specific genes, showing a wider range of gene regulation [20]. More recently, phosphorylation activity has been observed between trihelix factors and MAPK cascade genes [21], while binding activity was observed between trihelix factors and salicylic acid metabolism [12,22].

TTFs have not been thoroughly investigated in rhodophytes, though several investigations have provided some details. For example, [9] investigated a wide range of TFs across many Streptophyte algae, including six rhodophytes, which predicted no TTFs to be present, nor in the closely related glaucophyte *Cyanophora paradoxa*. Similarly, none were found in any rhodophytes (e.g., the microalgae *Galdieria sulphuraria* and macroalgae *Chondrus crispus*, *Gracilariopsis chorda*, *Porphyra umbilicalis*) following a broad-scale analysis of five different classes of plant TTFs [23]. A further study found no TTFs in a similar range of algae [8].

In contrast, a study of the microalga *Cyanidioschyzon merolae* 10D identified one TTF, but not in *C. crispus* or the microalga *Porphyridium purpureum* [7]. These findings are representative of TF research of rhodophytes in general, with most studies using limited species numbers (typically 3–10) [7,8,9]. However, the One Thousand Plant Transcriptome (1KP) [24] project utilised 28 rhodophyte transcriptomes, which was further interrogated by [25] to explore WUSCHEL-related homeobox (WOX) TFs, which have not been observed in rhodophytes [7,8,9].

The YABBY TF is another family of TFs with limited reported distribution in rhodophytes. YABBY TFs belong to a small family of TFs, which are a subset of the C2C2 set of TF families, along with Dof, CO-like, and GATA factors. Historically, YABBY factors are believed to have been exclusive to seed plants that regulate lamina outgrowth and leaf development [26,27]; however, more recent evidence has seen them identified in hornworts [28] and the green microalgae *Micromonas* [29], implying a more widespread distribution. In rhodophytes, they have been predicted in small numbers in seaweed from the class Bangiophyceae [8], as well as in the florideophyte seaweed *Calliarthron tuberculosum*, but not in other rhodophyte classes. YABBY TFs have also been connected to the flavonoid biosynthetic pathway, due to YABBY-binding motifs, and gene clustering with flavonoid pathway genes [27].

To provide a more comprehensive overview of TFs in rhodophytes, this study interrogated a wide range of rhodophyte genomes and transcriptome assemblies. To do this, we utilised assemblies that we previously collated and annotated in [30]. We then investigated the proportions of TFs across Rhodophyta. As previous studies had investigated TFs across a smaller range of algae from the classes Florideophyceae and Cyanidiophyceae, we hypothesised that TFs would retain a roughly similar proportion of TF families across our dataset. As our investigation identified TTF and YABBY factors where they have not previously been identified, we further investigated these TFs, with a focus on the TTFs identified in the Florideophyceae.

## 2. Results

### 2.1. Transcription Factor Predictions Were Consistent with Prior Studies

Based on transcription factors annotated with PlantTFDB v.5 online web server [1,31,32], we identified a total of 8424 individual TFs across all assemblies analysed, with 7235 predicted in rhodophytes. *Rhodosorus marinus* MMETSP0011 was excluded as an outlier due to an overly high number of protein annotations (as outlined in [30]), with 772 TF predictions, well above the three standard deviations from the mean of 383.3. The number of TF predictions varied from 30 to 239 (mean 95.65), though genomes typically had more TF predictions, with a mean of 113.7 and a median of 121, compared to the transcriptomes which had a mean and median of 74.7 and 47, respectively, with the transcriptomes from the One Thousand Plant Transcriptomes Project (1KP) typically having far fewer TFs than those from the Marine Microbial Eukaryotic Transcriptome Sequencing Project (MMETSP) (mean/median 74.7/47 compared to 160.5/166). Out of 58 annotated TF families, 38 were found in at least one assembly, while 25 were found in at least one rhodophyte assembly. The number of unique transcription factor families found in each assembly ranged from 11 to 30 (mean 16.23), with the mean in rhodophytes being 15.23.

**Figure 1:**
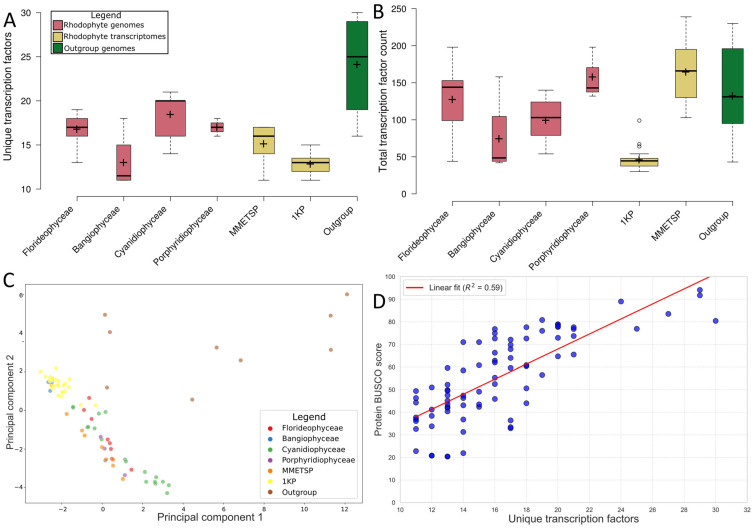
Distribution of TF annotations across rhodophytes. (**A**) Total counts of transcription factors inferred in all assemblies. (**B**) Unique transcription factor families inferred in different rhodophyte groups. (**C**) Principal component analysis of TF counts in all assemblies. (**D**) Scatterplot showing the correlation between the total number of unique transcription factor families found and the total BUSCO completion score.

Statistical analysis of the number of unique TFs with single-factor ANOVA and non-parametric Kruskal–Wallis (KW) analysis (Figure 1A) showed significant variation between groups (ANOVA: *p* = 7.37 × 10^−17^, F = 27.37, df = 79; KW: *p* = 0, df = 6, H = 50.03), however this variation was most prominent between the rhodophyte genomes and the 1KP samples, Bangiophyceae and other rhodophyte genomes and between the rhodophyte genomes and the outgroup genomes. This was due to the lower number of unique TFs predicted in the Bangiophyceae and 1KP samples, and the higher number of TFs predicted in the outgroup genomes. Between the florideophytes, cyanidiophytes, and porphyridiophytes, no significant variation was observed (ANOVA: *p* = 0.16, F = 1.95, df = 29; KW: *p* = 0.02, df = 2, H = 7.7849). The 1KP assemblies significantly differed from the other rhodophyte groups in terms of total TF inferences (*p* = 0, z = 9.68) (Figure 1B), with far fewer being inferred. This was reflected in the PCA of the TF counts (Figure 1C). The rhodophyte genome assemblies from the classes Florideophyceae, Cyanidiophycea, and Porphyridiophycae, as well as the MMETSP transcriptomes clustered broadly in the PCA, while the 1KP assemblies and the class Bangiophyceae genome assemblies clustered tightly together. The outgroup assemblies clustered separately from the rhodophytes, though *C. paradoxa* CCMP329 was the closest of the outgroup assemblies to the rhodophytes. This was reinforced with a linear regression showing that greater BUSCO completion rates had some correlation with a greater number of unique TF inferences (Figure 1D) (R^2^ = 0.59, *p* = 8.74 × 10^−17^), though this correlation did not extend to total TF inference counts (Supplemental Figure S1) (R^2^ = 0.2, *p* = 0.0007).

The total number of unique TF family predictions appeared to be largely reliant on the relative completeness of the assembly, though total counts were not. Total TF counts showed correlation with the total number of proteins in the assembly, but not assembly size, both with regard to all assemblies (R^2^ = 0.0171, *p* = 0.40) or only the genome assemblies (R^2^ = 0.0348, *p* = 0.098).

Other variations were observed between different clades. For most rhodophyte assemblies, the most common TFs inferred were C2H2 factors, with average predictions ranging from 27 to 44% of TFs inferred in Florideophyceae, Bangiophyceae, Porphyridiophyceae, and in the MMETSP and 1KP transcriptomes, but represented an average of 5.48% of TFs in Cyanidiophyceae, which was consistent with previous studies [5,7,8,9]. In contrast, in Cyanidiophyceae the most common TF type that was predicted were the MYB-related factors, with an average of 22.3% of all TF inferences in Cyanidiophytes. This was similar to the chlorophytes, however, which only had MYB-related TFs accounting for, on average, 12.1% of TF inferences in the included outgroup.

### 2.2. Putative Trihelix Factor Identified in Florideophyte

**Figure 2:**
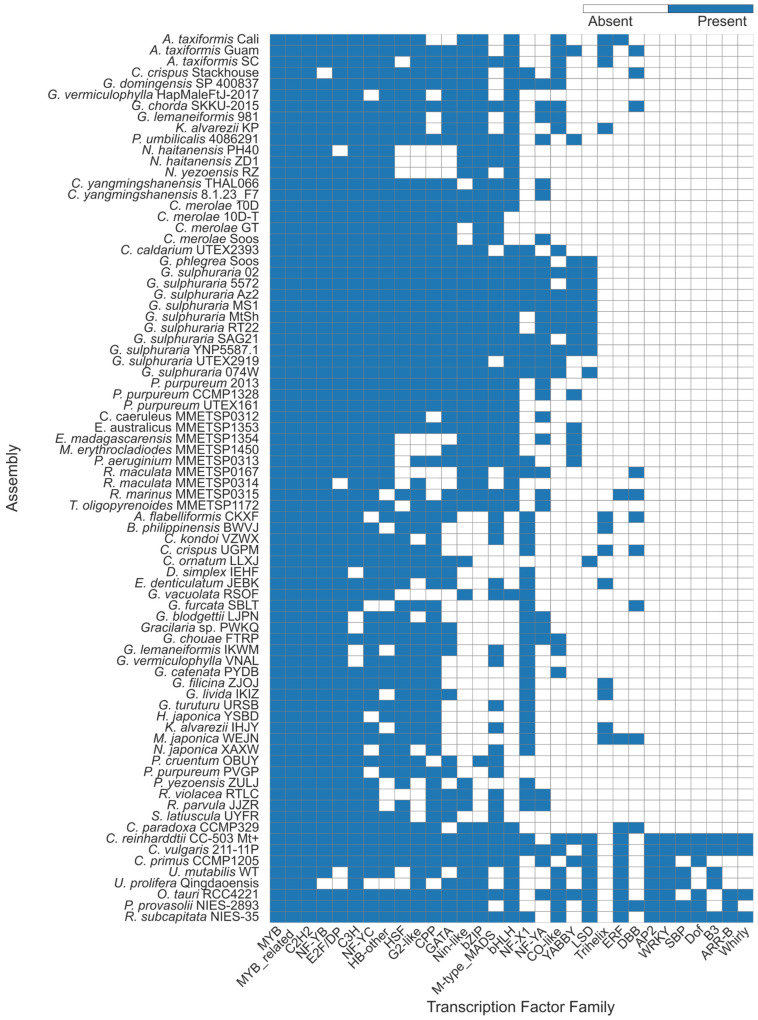
Presence (blue) and absence of TF families in each assembly based on PlantTFDB annotations.

**Figure 3:**
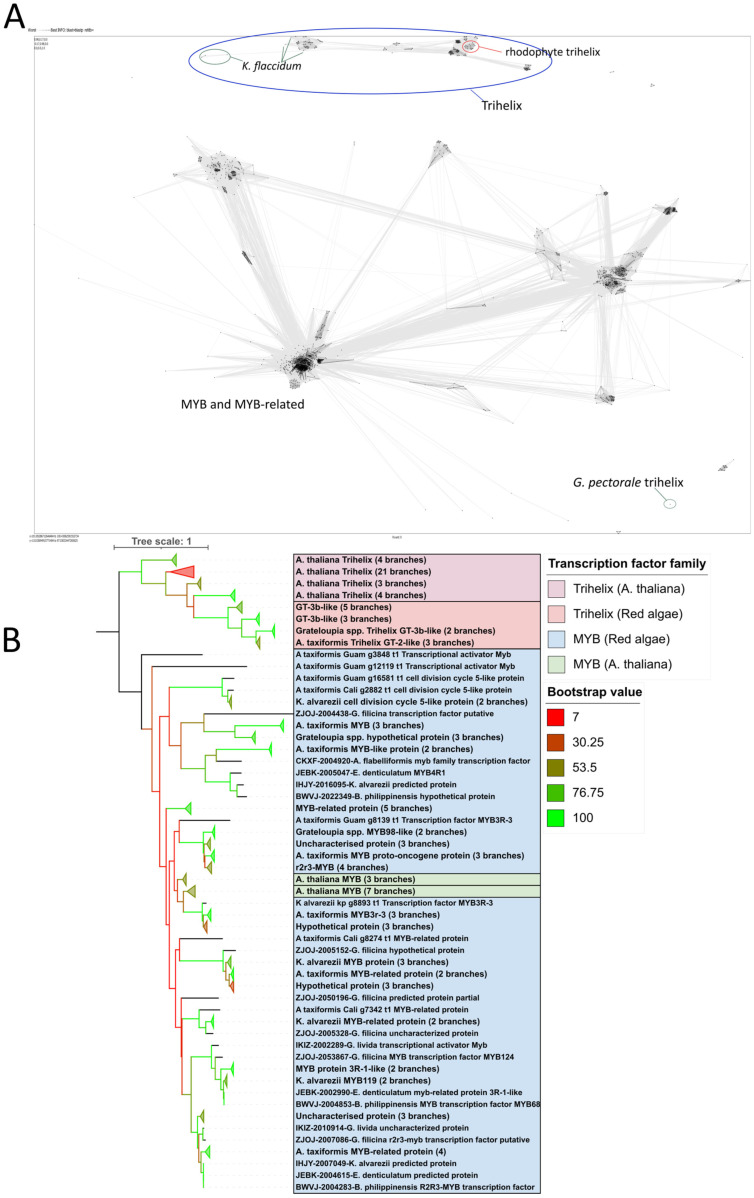
Phylogenetic analysis and clustering of TTF and MYB TFs in rhodophytes and *A. thaliana*. (**A**) CLANS clustering of the trihelix and MYB transcription factors. Darker lines represent stronger clustering between individuals. The trihelix cluster is circled in blue, while the rhodophyte trihelix factors are circled in red, and *K. flaccidum* and *G. pectoral* are circled in green. All other points represent MYB or MYB-related TFs. (**B**) Phylogenetic tree of rhodophyte trihelix proteins and a subset of trihelix and MYB proteins from rhodophytes and *A. thaliana*.

We further investigated the patterns of TF presence or absence to find clade or organism-specific TFs, which could correlate with specific biological phenomena. From this, we identified specific patterns between groups of rhodophyte assemblies. The 1KP assemblies were observed several times to be consistently missing TFs observed in the other rhodophyte categories, including the bHLH, bZIP, and Nin-like TFs (Figure 2, Supplemental Table S2), which was indicative of their smaller protein counts and shorter protein lengths [30], possibly due to the extensive trimming utilised in the 1KP experimental methods. Certain TFs were also noticeably present in specific assemblies. YABBY TFs were observed in 9 out of 11 *Galdieria* assemblies (all *Galdieria* spp. assemblies barring *G. sulphuraria* 074W and UTEX2919), but not in any of the other cyanidiophytes, and likewise was absent from the majority of other rhodophytes, though it was observed in four of the MMETSP samples, *P. umbilicalis*, and in the *Asparagopsis taxiformis* Guam (AtaGuam) assembly (Supplemental Table S2). Similarly, the LSD transcription factor was found in 10 of 11 *Galdieria* assemblies, and no other rhodophyte assemblies, barring *Chroodactylon ornatum* LLXJ. In Asparagopsis, TTFs were noticeably found in all three genome assemblies, as well as the *Kappaphycus alvarezii* genome, and from eight 1KP transcriptomes, including two from K. alvarezii IHJY, and another seven assemblies (Table 1). All 1KP transcriptomes showing the trihelix TF were florideophytes. One protein, AtaGuam g3848, was inferred to be a trihelix TF, but its protein annotation described it as a MYB TF, and clustered closer to the other MYB genes (Figure 3A). The Trihelix-annotated proteins had an average length of 352 aa in the genomes and 125.4 aa in the transcriptomes, due to the shorter open reading frames found in the 1KP transcriptomes.

BLASTp analysis using NCBI BLAST+ of the putative trihelix proteins returned a mix of plant Trihelix TFs and hypothetical protein annotations (Supplemental Table S3). Top percent identities ranged from 39.2 to 48.6% (mean 44.5%). In the genome-sourced proteins, E-values showed high certainty, often around 3 × 10^−7^ or better, but query coverage was low, often around 20–35%. The putative 1KP Trihelix proteins similarly had strong E-value scores, but also much higher coverage, though the total alignment lengths were similar. BLASTn analysis of the TTF genes against the rhodophyte assemblies only returned results of closely related species (Supplemental Table S4). *A. taxiformis* genes returned other *A. taxiformis* TTFs, *K. alvarezii* returned the 1KP *K. alvarezii* and the *B. philippinensis* TTF, and the *Grateloupia* spp. genes returned those of the same genus, and the *Chondrus crispus* TTF returned the *Mazzaella japonica* gene. The *Ahnfeltiopsis flabelliformis* and *Euchaeuma denticulatum* TTFs only returned themselves. Non-self BLAST hits had identity scores ranging from 82 to 100% (mean 94.09%), with E-values ranging from 1.77E-91 to 0 (mean 1.28 × 10^−92^). By contrast, BLASTp of the TTF proteins did return hits of all other TTF proteins (Supplemental Table S5), with identity percentages ranging from 55.8% to 100% (mean 68.2%), showing high conservation between assemblies of the order, family, or species, and much lower conservation across taxonomic boundaries.

To cross-validate the predicted TTF gene identities, we further supported the identity of these putative trihelix genes by querying them against the NCBI Conserved Domain Database [33,34,35] (Table 2), providing a more robust identification. The trihelix protein searches returned matches with the trihelix GT-1 gene, the 2A1904 superfamily of K^+^-dependent Na^+^/Ca^2+^-exchangers, and the Myb/SANT-like DNA binding domain. As the trihelix GT-1 protein binds the GT cis-element of rbcS-3A (ribulose bisphosphate carboxylase small subunit 3A), protein sequences for rbcS-3A were searched on NCBI Protein, which returned seven results; however, two were only a single amino acid in length and thus were excluded. BLAST analysis against the rhodophyte assemblies using an E-value cutoff of 2.0 returned BLAST results, but none for *Asparagopsis* or *Kappaphycus* (Supplemental Table S6).

As the trihelix and MYB TFs share conserved domains [12], the identity of the putative trihelix proteins was further investigated. All predicted rhodophyte trihelix, MYB, and MYB-related proteins were clustered, along with the correlating *Arabidopsis* proteins from PlantTFDB and all chlorophyte trihelix genes available on PlantTFDB, using CLANS (Figure 3A), which identified the trihelix-annotated proteins as a separate cluster. Only two chlorophytes (*Klebsormidium flaccidum* and *Gonium pectorale*) had trihelix TF annotations, both of which clustered further from the rhodophytes than the *Arabidopsis* proteins. This was further supported using phylogenetic analysis with a Neighbour Joining tree (Figure 3B, Supplemental File S1), which showed the MYB genes to be a separate outgroup from the trihelix proteins, which clustered together. A different pattern was observed when phylogeny was established using only the genomes, where the rhodophytes and *A. thaliana* clustered separately, each with their own separation between trihelix and MYB factors (Supplemental Figure S2). In both cases, however, the alignments only had lowly conserved regions and with relatively low bootstrap values. As YABBY TFs were similarly identified only in *Galdieria* spp., and likewise not widely predicted in previous studies, they were also investigated using this method (Supplemental Figure S3, Supplemental File S2). The majority of the YABBY genes clustered with those of *A. thaliana*, but the AtaGuam gene instead clustered with high mobility group (HMG) proteins when analysed with CLANS and formed a phylogenetic outgroup with itself. Alignment of the YABBY proteins with predicted GATA TF proteins (another C2C2 TF family) did not predict any conserved sites.

### 2.3. Trihelix Annotation Conserved Across Florideophyte Algae

We further compared the identified putative TTFs against similar genes to determine their evolutionary changes, and to determine whether these genes behaved as expected for a single gene, by analysing TTF nucleotide sequences. To generate groups of similar genes, we compared the 12 trihelix-possessing protein assemblies, as well as the *A. thaliana* TAIR10 assembly, with OrthoFinder v2.5.4. This generated a total of 15,597 orthogroups, of which 23 were single-copy orthologues (Supplemental File S3). The predicted trihelix genes were all found in a single orthogroup, OG0002371 (Supplemental File S4), in addition to a second *K. alvarezii* kp gene, g2440, and the *A. thaliana* protein NP_201147.2, both of which were annotated as metallo-beta-lactamase (MBL) family proteins. The CDS sequence for NP_201147.2 was found to be identical to the *A. thaliana* trihelix gene AT5G63420.1 from PlantTFDB, which was identified as a chimeric trihelix/MBL gene [14]. The trihelix orthogroup had excess genes trimmed, so that only one gene per individual was present. To infer gene diversity, we measured the tree length, phylogenetic diversity, symmetry, and mean entropy of the OG0002371 gene tree, as well as a selection of well-conserved genes (Table 3). OG0002371 had a genetic diversity of 4.06. By comparison, gene trees for typically well-conserved genes had similar values. To explore gene diversity between the TTF and MYB genes, we also measured trees of the orthogroup OG0002371 with an additional *A. thaliana* TTF or MYB gene added (Supplemental Table S7). These had similar genetic diversity values to the original OG0002371 tree and did not show significant variation between them (ANOVA: *p* = 0.19, F = 1.79, df = 1; KW: *p* = 0.30, H = 1.08).

**Figure 4:**
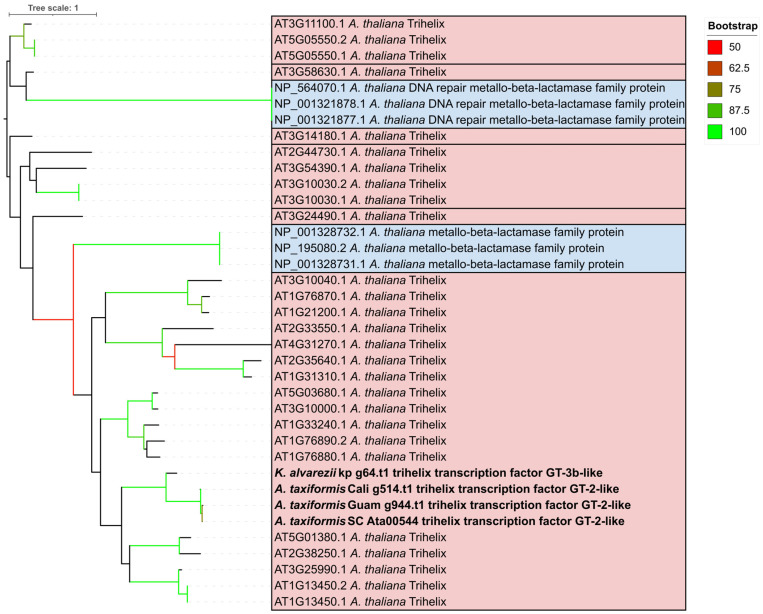
Neighbour-joining tree of trihelix and MBL proteins. Tree was rooted at the midpoint. Predicted protein family annotations are marked. Rhodophyte genes with predicted trihelix elements are in bold. Genes from the 1KP transcriptomes were excluded due to poor alignments.

To further compare against the generated orthogroups, custom orthogroup variants of the trihelix orthogroup were created by adding the *A. thaliana* trihelix CDS sequences retrieved from PlantTFDB. To further support the delineation of the predicted TTF and MBL genes, a further phylogenetic tree was inferred using all the trihelix proteins, as well as all MBL-annotated proteins from the *A. thaliana* assembly (Figure 4, Supplemental File S5). As the initial alignment was of poor quality, only genes from the genomes were used. The rhodophyte trihelix proteins clustered with the *A. thaliana* trihelix proteins, though there was some overlap between the trihelix and MBL genes, and bootstrap values were relatively low.

### 2.4. Protein Modelling and Motif Prediction

**Figure 5:**
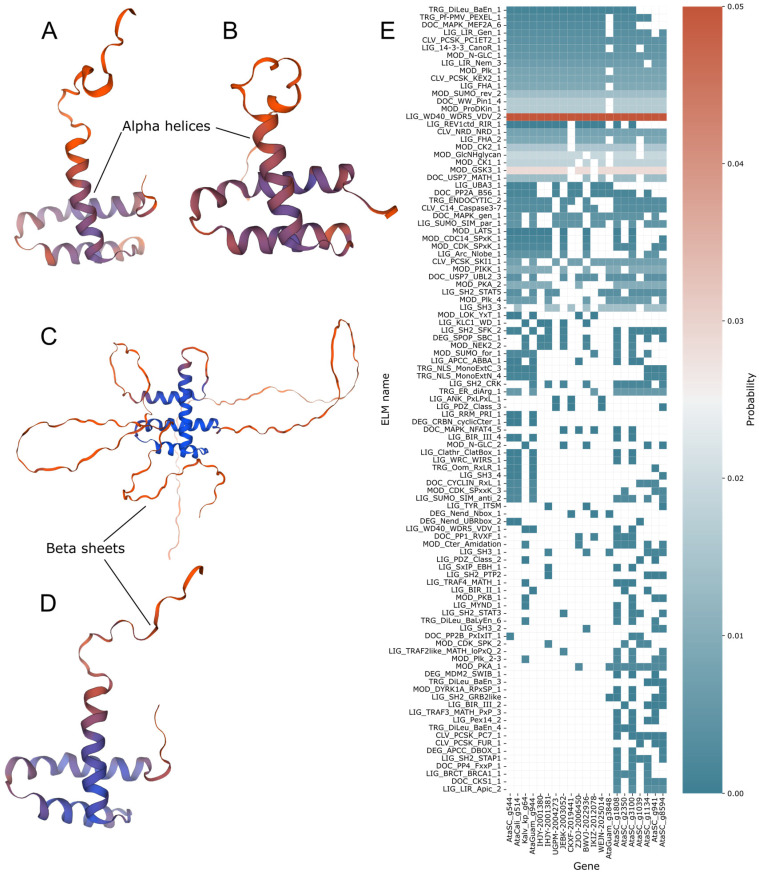
Protein structure and motif predictions for TTF proteins. (**A**) *A. taxiformis* SC Ata00544 TTF models 1 and (**B**) 2. (**C**) *A. thaliana* AT1G13450.3 models 1 and (**D**) 2. (**E**) Heatmap of predicted probabilities of linear motifs for rhodophyte trihelix transcription factors, which correlates to the strength of amino acid regular expression values, as well as a selection of MYB factors. Columns were clustered using average linkage and Euclidean distance measurement. ELMs with two or fewer predictions were excluded. White cells represent null values where no predictions were inferred.

To further support the putative TTF annotation, we investigated their protein structures using protein modelling via the SWISS-MODEL programme [36] (Figure 5A–D). The top protein models correlated best with *Arabidopsis* GT-1 proteins (templates 2jmw.1.A (Figure 5A) and 2ebi.1.A (Figure 5B)). For all *A. taxiformis* trihelix genes (Figure 5A,B), both coverage and sequence identity were low, with a GMQE of 0.08 and a QMeanDisCo Global score of 0.55 ± 0.09 in the *A. taxiformis* SC (AtaSC) trihelix. The highest confidence was observed in the lower helices of the proteins near the N-terminus. To compare, the *A. thaliana* TTF protein AT1G13450.3 was also analysed. *A. thaliana* model 1 (template Q9FX53.1.A) (Figure 5C) showed a much larger protein model, with extensive beta-sheets, but with a core of three alpha-helices consistent with the other trihelix models, barring an additional alpha helix. *A. thaliana* model 2 (template 2jmw.1.A) (Figure 5D), however, was much more similar in structure to the *A. taxiformis* TTF proteins. As expected, both *A. thaliana* models showed much higher similarity scores, due to the evolutionary distances between *A. taxiformis* and *A. thaliana*.

We further supported the differentiation between the rhodophyte TTF and MYB factors by searching for binding motifs (Supplemental Table S8). Motif structures for the rhodophyte trihelix TF were further investigated using the ELM resource [37,38] (Figure 5E), as well as a small selection of MYB TFs from AtaSC. Only motifs with predictions in at least two assemblies were included to prevent false positives. AtaGuam g3848 was missing several motifs present in all other trihelix-annotated proteins, though it was also distinct from example MYB proteins from AtaSC. Predicted motifs all had probabilities assigned by the ELM tool, which annotates these motifs based on their amino acid regular expression patterns, with higher values indicating a potentially more degenerate protein [37,38,39], and which by default uses a probability cutoff of 0.4. E-values were based on motif regular expression and do not vary between sequences. A probability threshold of 0.01 was adopted for positive predictions, and 0.001 for high-confidence predictions. Four ELMs showed strong probabilities and were present in all or almost all trihelix-annotated genes. These ELMs had probabilities ranging from 0.0026 to 4.78 × 10^−4^. However, there were 27 probability predictions with scores of 1 × 10^−4^ or under, though these inferences were not predicted in any more than five of the 13 trihelix-annotated genes. As trihelix TFs have been shown to interact with MAPK proteins in other plants [21,22], we searched for MAPK binding. The most relevant MAPK ELM inferred in the TTF proteins was the ELM DOC_MAPK_MEF2A_6, a MAPK-docking motif, and was found in all 13 trihelix-annotated proteins. However, it was a weaker prediction, with a probability score of 0.0022, which exceeded the 0.01 threshold for general predictions, but did not reach the threshold for a high-quality prediction.

## 3. Discussion

Our understanding of transcription factors in rhodophytes has until recently been limited and typically restricted to a small range of rhodophytes. This study investigated the proportions of TFs across the phylum Rhodophyta, utilising a wide range of rhodophyte assemblies, and in doing so, extended the range of known TF predictions than previously investigated.

### 3.1. Overall Transcription Factor Proportions Supported Previous Studies

The overall patterns of TF composition generally reflected prior research, but with a larger range of data. In the present study, of all TF families, the C2H2 factors were observed to be in the largest proportion in non-cyanidiophyte algae (20–40%), which aligns with that predicted by prior studies [5,7,8,9]. C2H2 factors were in a much smaller proportion in cyanidiophyte algae. Those TF families identified as absent (e.g., AP2 and WOX factors in all rhodophytes) also supported prior evidence [5,7,8,9,25,40], potentially due to their evolution in plants after Rhodophyta diverged, or otherwise due to gene loss [40]. The three ERF factors identified in *A. taxiformis*, *Rhodosorus marinus*, and *Mazzaella japonica* were potentially false positives, as no other related organisms reported them, and they have been previously reported as being completely absent from rhodophytes. This research also supported previous observations that genome size did not have a direct relationship with TF family count [41], though the correlations between protein counts and BUSCO completion scores with unique TF families point to a relationship with assembly completeness. However, the inferred correlations only had moderate strength, with confounding variables such as assembly fragmentation and variable sequencing and assemblage methodologies being possible causes, with drastic differences in metrics such as contig numbers and N50 scores between different assemblies.

### 3.2. Trihelix and YABBY TFs Were Predicted Contrary to Previous Evidence

Several TF families were notably predicted as present, contrary to prior studies, including YABBY and TTFs; YABBY factors were observed in *Galdieria* spp., while TTFs were present in several florideophyte algae. Trihelix factors had not previously been observed in florideophyte algae, with these factors not identified in any rhodophytes [8,9,23]. Interestingly, TTFs were once predicted in a single *C. merolae* assembly [7], which was not reflected in this dataset. However, there have been previous studies showing support for these findings. Collén et al. [42] showed a SANT-subfamily MYB transcription factor in their sequencing of the *C. crispus* genome, while the trihelix TF was also previously predicted using PlantTFDB in *C. crispus* by, and likewise not in *P. purpureum*, *G. sulphuraria*, or *C. merolae* [43]. The Phycocosm dataset currently has a single MYB/SANT TF predicted in *Rhodosorus marinus* [44]. It should be noted that in the assemblies previously predicted not to have TTFs (Supplemental Table S9), they were also not observed in this dataset. This may imply that these putative TTFs are present in a limited clade or population of rhodophytes, though further testing will be required to verify this. The limited quality of some of the rhodophyte assemblies available does limit the efficacy of widespread in-depth analysis, particularly with the 1KP assemblies having far fewer and much shorter protein annotations.

The putative TTF gene showed a highly conserved region within the rhodophytes, and a small number of conserved nucleotides between both the rhodophytes and *A. thaliana*, as well as with MYB and MYB-related factors, which mirrors evidence of high conservation of the traditional MYB domain [45]. Overall, gene conservation between different rhodophytes was low. Most groups of putative TTFs that did not return BLAST results were separated at the order level, though the difference between the *Chondrus*/*Mazzaella* and *Kappaphycus*/*Betaphycus* groups was at the family level. Protein annotations also classified some as either GT-2 or GT-3 TTF families, though this is not certain, with the variability likely due to misannotation or gene family expansion and would need further investigation to confirm. Protein motif analysis provided some support to this claim, with a consistent MAPK-binding motif observed across all putative TTF proteins; however, the significance was low. While this may indicate a false positive, it is possible that it shows degenerate motifs. Similarly, protein modelling relied on comparing against *A. thaliana models*, which resulted in consistently low-quality predictions, due to the low sequence similarity between the *A. thaliana* and rhodophyte TTF proteins.

Thus, further investigation, such as the investigation of further assemblies or in vitro gene characterisation, would be required to determine if these were truly different TTF families, though, or if these genes just showed high variability between assemblies. Overall, we believe there is reasonable evidence that the putative TTFs were not MYB or MYB-related factors. Furthermore, the evidence suggests that these are not merely MBL proteins. Yet, further evidence would be required to determine if these are true TTF proteins, or if they are another MYB or MBL orthologue, or perhaps even chimeric proteins, such as a TTF/MBL chimera, as have been previously identified [14,46]. Thus, a more thorough investigation of these proteins, including experimental validation, would be required to truly confirm whether these are true TTFs, degraded TTF or TTF-like proteins, or another MYB or MYB-like factor. The predicted TTF gene AtaGuam g3848, which was annotated as an MYB gene in other annotations, was almost certainly a false prediction, with the false prediction likely caused by the overlap of domains, as AtaGuam g3848 had low similarity to the predicted TTFs. The protein likely belonged to either a MYB or MYB-related family, as evidenced by the consistent differences to the putative TTFs in the CDD, Orthofinder, and protein modelling analyses. Interestingly, CLANS clustering put the rhodophyte TTF genes closer to *A. thaliana* than to the charophyte algae. Evidence has previously shown that green algae, in particular chlorophytes, have unique TF lineages that cause them to have distinct TF profiles to Rhodophyta, Haptophyta, and the Stramenopiles [5].

The presence of YABBY TFs in *Galdieria* spp. most strongly contradicts previous studies, though the other predictions (and lack thereof) mirror previous studies. As with previous studies, the YABBY TF was observed in bangiophyte algae [7,8,9], though not comprehensively. Researchers did not find it in *P. purpureum* [8], which, in conjunction with these results, suggests that the TF may only be observed sporadically across different strains or lineages, though further research would be needed to assert that. Two studies observed it in *Calliarthron tuberculosum*, but not *C. crispus* [8,9]. It is possible that this factor is present (or even widespread) in the subclass Corallinophycidae, from which *C. tuberculosum* is derived from, but none of the annotated assemblies in this dataset were sourced from that subclass. In the cyanidiophyte algae, there was a strong split between Galdieria and the other cyanidiophytes, including even *C. caldarium*, which supports previous results [7,8,47]. Though previous studies did not report YABBY TFs in *Galdieria*, two *Galdieria* spp. assemblies (*G. sulphuraria* Az2 and *G. yellowstonensis*) were annotated with it in the Phycocosm database, though the remaining *Galdieria* assemblies did not possess it. Interestingly, all the *Galdieria* spp. assemblies that had the YABBY TFs predicted came from a single study [48].

There are two potential explanations for the presence of YABBY TFs in Cyanidiophyceae and Bangiophyceae. One potential explanation is that this TF was present in a common ancestor but subsequently lost by most classes. A similar situation has been observed with other C2C2 factors, with GATA families predicted to be present in the common ancestor of algae and subsequently lost in the Stramenopiles [5]. The subsequent loss of this TF in the Cyanidiales could potentially be explained by the substantial gene losses they have experienced [48,49]. Alternatively, horizontal gene transfer is a possible explanation for its presence in *Galdieria*, with microalgae such as *G. sulphuraria* having significant elements of its genome that have sources attributed to horizontal gene transfer [48], while horizontal gene transfer has also been investigated as the potential source for bangiophyte macroalgae [50]. As with the TTF predictions, experimental validation, such as functional characterisation or qPCR, would be required to confirm whether YABBY TFs are truly present in *Galdieria*, and to what extent.

### 3.3. Putative TF Predictions May Have Impacts on Secondary Metabolism

TFs have a broad range of regulatory impacts, which can greatly affect a large range of different biological functions, which can have significant impacts on how organisms may be utilised. Algae have a sizeable industry built around their exploitation for secondary metabolites, and as such, an understanding of how different TFs impact their secondary metabolism is important.

The TTF family of proteins has various functions. The GT2-like 1 (GTL1) proteins have been found to be associated with innate immunity through interactions with the MAPK cascade and positively regulated salicylic acid biosynthesis [22], but also negatively regulates growth in plants [14]. Likewise, the MAPK signalling pathway has been shown to interact with genes from both the MBL and MYB families [20]. In humans, [51] showed that silencing of the *zDHHC20* MBL gene led to hyperactivation of the MAPK pathway. Similar proteins have also been shown to interact with the MAPK pathway, with knockdown of the ADAM metallopeptidase domain 10 leading to suppression of the MAPK pathway [52]. Chimeric genes of both TTF and MBL genes have also been identified in rice [46]. Similarly, TTFs have been observed to regulate other metabolic pathways. In the flowering plant *Pogostemon cablin* TTF overexpression was observed to negatively regulate the biosynthesis of the sesquiterpenoids such as patchoulene, trans-caryophylene, α-bulnesene and α-guaiene [53]. Thus, it is possible that TTF expression in rhodophytes could significantly impact the regulation of secondary metabolites such as terpenoids. It is also possible that the putative TTFs identified in the current study perform different functions in rhodophytes compared to land plants, or that these proteins represent a related orthologue of the plant TTF, which would require further research to determine. Similarly, further research would be required to identify any phylogenetic or ecological correlations between species containing TTFs, due to the limited genomic data available.

It is not yet known how YABBY TFs would affect microalgae, though colony growth or cell development is a likely regulatory target. However, the role of YABBY TFs in other plants can be used to inform potential regulatory roles. In particular, the connection between YABBY TFs and flavonoid biosynthesis [27] would suggest a potential role in microalgae. Microalgae are known to produce a range of flavonoids, phenolic compounds which can have antiviral activity [54]. For example, *G. sulphuraria* has been shown to have high phenol content [55], while *P. purpureum* has been shown to possess other flavonoids, including apigenin [56]. However, in our previous investigation of rhodophyte metabolic pathways, we found that of the three-flavonoid biosynthesis-associated pathways in KEGG, two were completely absent in all rhodophytes (M00138 and M00940), while the third (M00137) was incomplete or absent [30]. Putative in silico predictions for microalgal phenol biosynthesis pathways have been predicted but have yet to be experimentally validated [57].

## 4. Materials and Methods

### 4.1. Identification of TFs Across Rhodophyte Assemblies

Rhodophyte genome and transcriptome assemblies that were collated and annotated in [30] were utilised for this study. A total of 81 assemblies were used, including 72 rhodophytes and an outgroup of 8 chlorophytes and one glaucophyte (Supplemental Table S1). In the rhodophytes, 34 were genome assemblies, while 38 were transcriptome assemblies originally sourced from the One Thousand Plant Transcriptome (1KP) project [24] and the Marine Microbial Eukaryotic Transcriptome Sequencing Project (MMETSP) [58]. TFs were predicted using the PlantTFDB v.5 online web server [1,31,32], as it contains TF data from a wide range of plants, including land plants and algae from the phyla Chlorophyta and Charophyta. TFs were categorised into families according to PlantTFDB.

Overall, TF patterns were compared between assemblies by grouping the assemblies into seven groups: the rhodophyte classes for the genome assemblies, the 1KP and MMETSP transcriptomes, and the outgroup genomes. The 1KP transcriptomes are predominantly composed of algae from the class Florideophyceae, while the MMETSP transcriptomes include samples from the classes Compsopogonophyceae, Porphyridiophyceae, Stylonematophyceae, and Rhodellophyceae. The transcriptomes were analysed separately from the genomes due to consistent differences in the protein annotations, as observed in [30]. Rates of presence between these groups were then analysed using Principal Component Analysis (PCA).

### 4.2. Phylogenomic Analysis of Putative Trihelix Transcription Factors

All Trihelix, MYB, and MYB-related proteins were retrieved from all rhodophyte assemblies with trihelix transcription factors (TTF) annotations. These proteins were clustered against the trihelix annotated proteins, as well as all MYB and MYB-related proteins from assemblies with trihelix annotations, as well as all trihelix, MYB, and MYB-related green algal and *A. thaliana* proteins from PlantTFDB, using the CLANS toolkit [59,60,61,62], as well as phylogenetic analysis using the MAFFT server [63,64,65] using the G-INS-1 model, with trees inferred using the Neighbour Joining model with at least 1000 bootstraps. Similarities between genes were established using BLAST searches against the dataset used in this study (see data availability statement) using NCBI BLAST+ v2.12.0. Phylogenomic comparisons were also performed using nucleotide sequences. First, putative genes were identified by using the programme OrthoFinder v2.5.4 [66,67,68] on the protein assemblies of the relevant assemblies as well as the *Arabidopsis thaliana* TAIR10 genome assembly. OrthoFinder was run using standard options, with gene search performed with DIAMOND [69] and similarities calculated using DendroBLAST [70]. Single-copy orthologues and orthologues containing other relevant genes were extracted from the OrthoFinder results, and the corresponding coding sequences retrieved. Where an assembly had more than one sequence in an orthologue, the longest sequence was kept. These orthologues were then aligned and had phylogenies inferred using MAFFT [71] and RAxML-NG v.0.9.0 [72], respectively. Phylogeny was inferred using the RAxML-NG’s ‘--all’ argument, using the GTR+G model (for CDS sequences) or the GTT+G model (for protein sequences), and with a bootstrap value of 250. Phylogenetic trees were then compared using the ETE3 Toolkit v3.1.3 [73] Python library to determine maximum tree length.

### 4.3. Protein Modelling and Motif Analysis

Identities were further established using protein modelling via the SWISS-MODEL online server [36]. This was supported using motif searches using the Eukaryotic Linear Motif (ELM) resource [37,38] and the NCBI Conserved Domains Database [33,34,35], to ascertain identity by correlating known motifs and domains. For the ELM predictions, a probability cutoff of 0.01 was utilised for general predictions, and 0.001 for high-confidence predictions. Motifs were predicted in proteins with TTF predictions, as well as a small selection of MYB genes from AtaSC for comparison.

### 4.4. Statistical Analysis

Statistical analysis on total and unique TF prediction counts, grouped either by class for genome assemblies and source project for transcriptome assemblies, was performed using single-factor ANOVA and two-tailed *t*-tests for parametric tests, and Kruskal–Wallis and Mann–Whitney tests for non-parametric analysis. Normality was tested with Shapiro–Wilk tests, though both parametric and non-parametric results were reported. The statistical significance of the differences between the 1KP and other TF predictions were evaluated using a two-tailed Z-test. Total counts of each family of TF were then analysed and clustered by genome class and transcriptome source using PCA.

## 5. Conclusions

Transcription factors are important proteins found throughout all eukaryotic organisms that regulate gene transcription. While previous experiments and datasets have investigated the TFs of rhodophytes, this study represents the broadest investigation of rhodophyte TFs to date. Overall, the results demonstrated that the TF overall numbers were largely consistent with previous studies. However, our data found two TF families that were not reported in previous studies. YABBY TFs, which are associated with lipid and flavonoid biosynthesis, were predicted across *Galdieria* assemblies, while putative TTFs were predicted in several florideophytes, including *A. taxiformis*.

This study used phylogenomics and protein structure analysis to investigate transcription factors in rhodophytes and identified a putative TTF in *A. taxiformis* and across several other florideophyte assemblies. Phylogenomic investigation found that the putative TTFs showed highly conserved regions with related MYB TFs and metallo-β-lactamases, but clustered distinctly away from them, and showed greater similarity to the TTFs of *A. thaliana*. This was supported by protein modelling and motif prediction which identified motifs associated with TTFs and MYB factors, as well as a protein structure most similar to other TTFs, though with low certainty due to high phylogenetic distance.

This research opens avenues of further research into how *A. taxiformis* and other rhodophytes regulate genes associated with important metabolic processes. Further research will be required to fully confirm the identity of the putative trihelix factors and their functional characteristics in rhodophytes, such as through functional characterisation or gene knockouts, while similar confirmation will be required for the putative YABBY TFs in *Galdieria*.

## Figures and Tables

**Table 1:** Trihelix-annotated proteins inferred in rhodophyte assemblies.

Query	Name	Accession	Interval	E-Value	Coverage %
A_taxiformis_SC_g544.t1	GT1	cd12203	9–75	1.12 × 10^−18^	14.44201
A_taxiformis_SC_g544.t1	2A1904 super family	cl36772	255–443	4.59 × 10^−3^	41.13786
A_taxiformis_Cali_g514.t1	GT1	cd12203	9–75	7.88 × 10^−19^	14.44201
A_taxiformis_Cali_g514.t1	2A1904 super family	cl36772	255–443	1.90 × 10^−4^	41.13786
K_alvarezii_kp_g64.t1	Myb_DNA-bind_4	pfam13837	9–94	8.37 × 10^−16^	24.14773
K_alvarezii_kp_g64.t1	2A1904 super family	cl36772	202–342	3.46 × 10^−5^	39.77273
A_taxiformis_Guam_g944.t1	GT1	cd12203	6–72	1.11 × 10^−18^	14.53744
scaffold-IHJY-2001380-Kappaphycus_alvarezii	Myb_DNA-bind_4	pfam13837	11–96	1.98 × 10^−15^	81.73077
scaffold-IHJY-2001381-Kappaphycus_alvarezii	Myb_DNA-bind_4	pfam13837	79–164	4.60 × 10^−18^	49.4186
scaffold-UGPM-2004273-Chondrus_crispus	GT1	cd12203	18–82	3.39 × 10^−17^	60.37736
scaffold-JEBK-2003052-Eucheuma_denticulatum	GT1	cd12203	81–145	2.81 × 10^−17^	60.37736
scaffold-CKXF-2019441-Ahnfeltiopsis_flabelliformis	GT1	cd12203	1–65	4.54 × 10^−16^	71.91011
scaffold-ZJOJ-2006450-Grateloupia_filicina	GT1	cd12203	38–104	2.21 × 10^−15^	51.9685
scaffold-BWVJ-2022936-Betaphycus_philippinensis	Myb_DNA-bind_4	pfam13837	73–158	1.35 × 10^−18^	51.20482
scaffold-IKIZ-2012078-Grateloupia_livida	GT1 super family	cl23759	1–65	7.35 × 10^−15^	72.72727
scaffold-WEJN-2025014-Mazzaella_japonica	GT1	cd12203	18–82	3.39 × 10^−17^	60.37736
A_taxiformis_Guam_g3848.t1	SANT super family	cl21498	17–95	8.67 × 10^−6^	65.54622
A_taxiformis_Guam_g3848.t1	SANT	smart00717	84–119	4.10 × 10^−3^	29.41176

**Table 2:** NCBI conserved domains database matches with the TTF annotated genes, as well as the probable false positive *A. taxiformis* Guam g3848.t1.

Query	Name	Accession #	Interval	E-Value
Ata00544	GT1	cd12203	9–75	1.12 × 10^−18^
Ata00544	2A1904 super family	cl36772	255–443	4.59 × 10^−3^
A_taxiformis_Cali_g514.t1	GT1	cd12203	9–75	7.88 × 10^−19^
A_taxiformis_Cali_g514.t1	2A1904 super family	cl36772	255–443	1.90 × 10^−4^
K_alvarezii_kp_g64.t1	Myb_DNA-bind_4	pfam13837	9–94	8.37 × 10^−16^
K_alvarezii_kp_g64.t1	2A1904 super family	cl36772	202–342	3.46 × 10^−5^
A_taxiformis_Guam_g944.t1	GT1	cd12203	6–72	1.11 × 10^−18^
scaffold-IHJY-2001380-Kappaphycus_alvarezii	Myb_DNA-bind_4	pfam13837	11–96	1.98 × 10^−15^
scaffold-IHJY-2001381-Kappaphycus_alvarezii	Myb_DNA-bind_4	pfam13837	79–164	4.60 × 10^−18^
scaffold-UGPM-2004273-Chondrus_crispus	GT1	cd12203	18–82	3.39 × 10^−17^
scaffold-JEBK-2003052-Eucheuma_denticulatum	GT1	cd12203	81–145	2.81 × 10^−17^
scaffold-CKXF-2019441-Ahnfeltiopsis_flabelliformis	GT1	cd12203	1–65	4.54 × 10^−16^
scaffold-ZJOJ-2006450-Grateloupia_filicina	GT1	cd12203	38–104	2.21 × 10^−15^
scaffold-BWVJ-2022936-Betaphycus_philippinensis	Myb_DNA-bind_4	pfam13837	73–158	1.35 × 10^−18^
scaffold-IKIZ-2012078-Grateloupia_livida	GT1 super family	cl23759	1–65	7.35 × 10^−15^
scaffold-WEJN-2025014-Mazzaella_japonica	GT1	cd12203	18–82	3.39 × 10^−17^
A_taxiformis_Guam_g3848.t1	SANT super family	cl21498	17–95	8.67 × 10^−6^
A_taxiformis_Guam_g3848.t1	SANT	smart00717	84–119	4.10 × 10^−3^

**Table 3:** Phylogenetic tree statistics for nucleotide trees inferred using OrthoFinder results.

Orthologue	Description	Tree Length	Phylogenetic Diversity	Mean Entropy
OG0001075	20S core proteasome subunit alpha 1	2.04459	3.81446	0.712415
OG0002371	Trihelix	1.737687	4.060484	0.789495
OG0001145	CCR4-NOT transcription complex subunit	1.822181	4.154696	0.965758
OG0003140	T-complex protein 1 subunit	2.460099	4.209469	0.589745
OG0003182	DNA-directed RNA polymerase	1.94762	4.402117	0.682362
OG0003030	GTP-binding family protein	2.570532	4.42165	0.765911
OG0001141	Pyruvate dehydrogenase E1 component alpha subunit	2.500867	4.43349	0.842321
OG0001163	Signal recognition particle, SRP54 subunit protein	2.582355	4.488274	0.940031
OG0001156	vacuolar ATP synthase subunit A	2.093928	4.580014	0.893024
OG0002704	carbamoyl phosphate synthetase B	2.904098	4.607873	0.80962
OG0001079	Ubiquitin thioesterase OTU1	2.502898	4.654698	0.874793

## Data Availability

The original contributions presented in this study are included in the Supplementary Materials. The original data presented in this study is also available at the University of the Sunshine Coast Research Bank at https://doi.org/10.25907/00954.

## Supplementary Materials

The following supporting information can be downloaded at: https://www.mdpi.com/article/10.3390/plants14203143/s1, Figure S1: Scatter plot comparing total TF counts to protein BUSCO scores; Figure S2: Phylogeny of MYB and TTFs from *A. thaliana* and rhodophyte genomes. Rhodophytes are shown in bold; Figure S3: Phylogenomic analysis of YABBY TFs; Table S1: Counts of TF predictions for each assembly; Table S2: Counts of each TF family for each assembly; Table S3: NCBI BLASTp results of each putative TTF; Table S4: Custom BLASTn results of each putative TTF; Table S5: Custom BLASTp results of each putative TTF; Table S6: Custom BLASTp results of the rbcS-3A protein; Table S7: Phylogenetic tree analysis statistics; Table S8: ELM results; Table S9: Phylogenetic tree analysis statistics; File S1: Alignment of TTF and MYB protein sequences; File S2: TTF and YABBY protein and nucleotide sequences; File S3: OrthoFinder results for TTF-containing assemblies; File S4: Nucleotide alignment corresponding to orthologue OG0002371; File S5: Sequence alignment of TTF and MBL protein sequences.

## Abbreviations

The following abbreviations are used in this manuscript:

1KP	One Thousand Plant Transcriptomes Project
AtaCali	*Asparagopsis taxiformis* Cali
AtaGuam	*Asparagopsis taxiformis* Guam
AtaSC	*Asparagopsis taxiformis* Sunshine Coast
BLAST	Basic Local Alignment Search Tool
BUSCO	Benchmarking Universal Single Copy Orthologs
CLANS	Cluster ANalysis Sequences
KW	Kruskal–Wallis
MAPK	Mitogen-activated protein kinase
MBL	Metallo- β-lactamase
MMETSP	Marine Microbial Eukaryotic Transcriptome Sequencing Project
PlantTFDB	Plant Transcription Factor Database
TF	Transcription factor
TTF	Trihelix transcription factor
WOX	WUSCHEL-related homeobox
