# Supplementary figures and images for: Transcription Factor Analysis of Rhodophytes Suggests Trihelix Transcription Factors Across the Florideophyceae
Source: Plants (Basel). 2025 Oct 12;14(20):3143. doi: 10.3390/plants14203143 (PMC12567131; doi:10.3390/plants14203143)

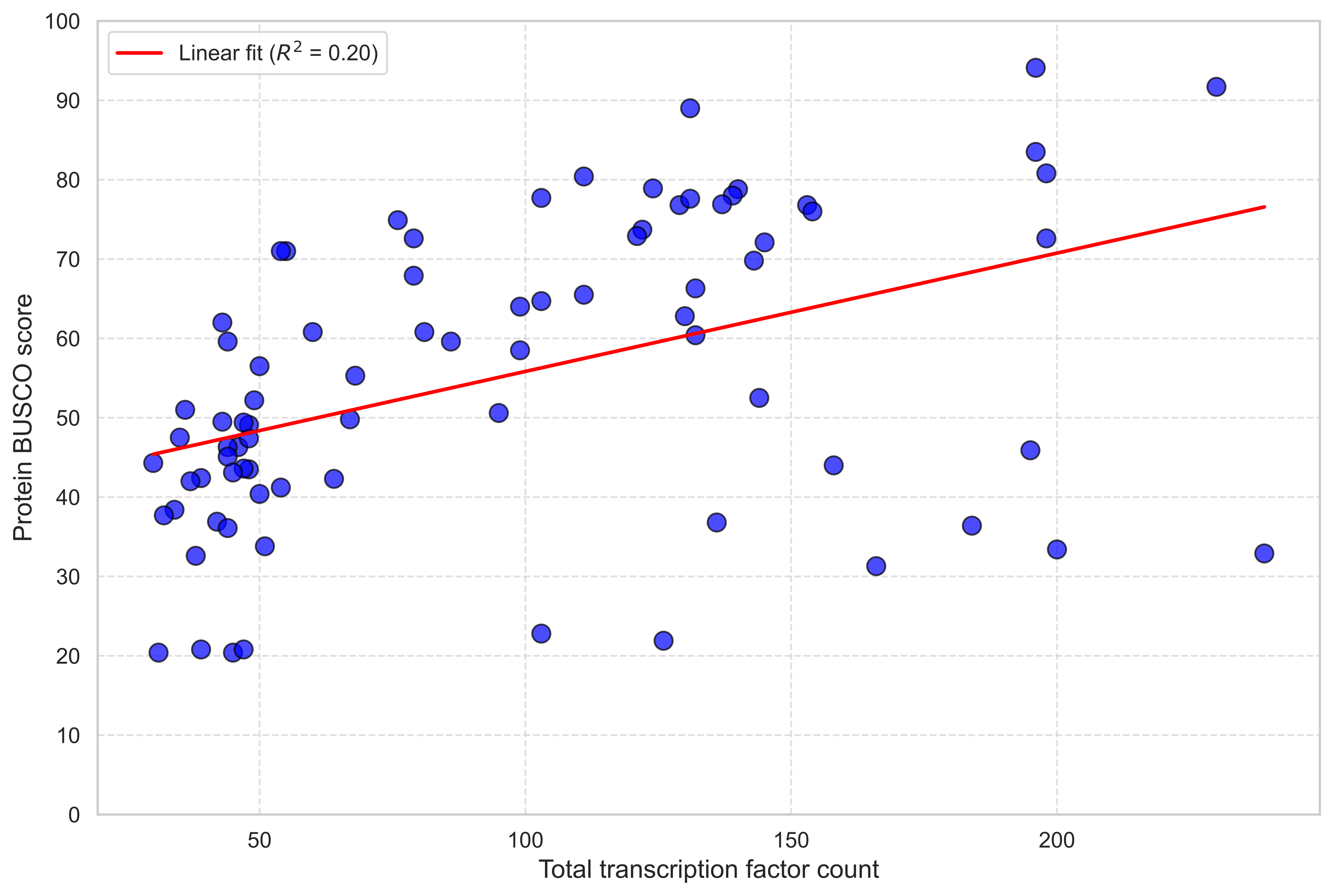

Supplement: Supplementary file 1 [file plants-14-03143-s001.zip › Figure S1.tiff]

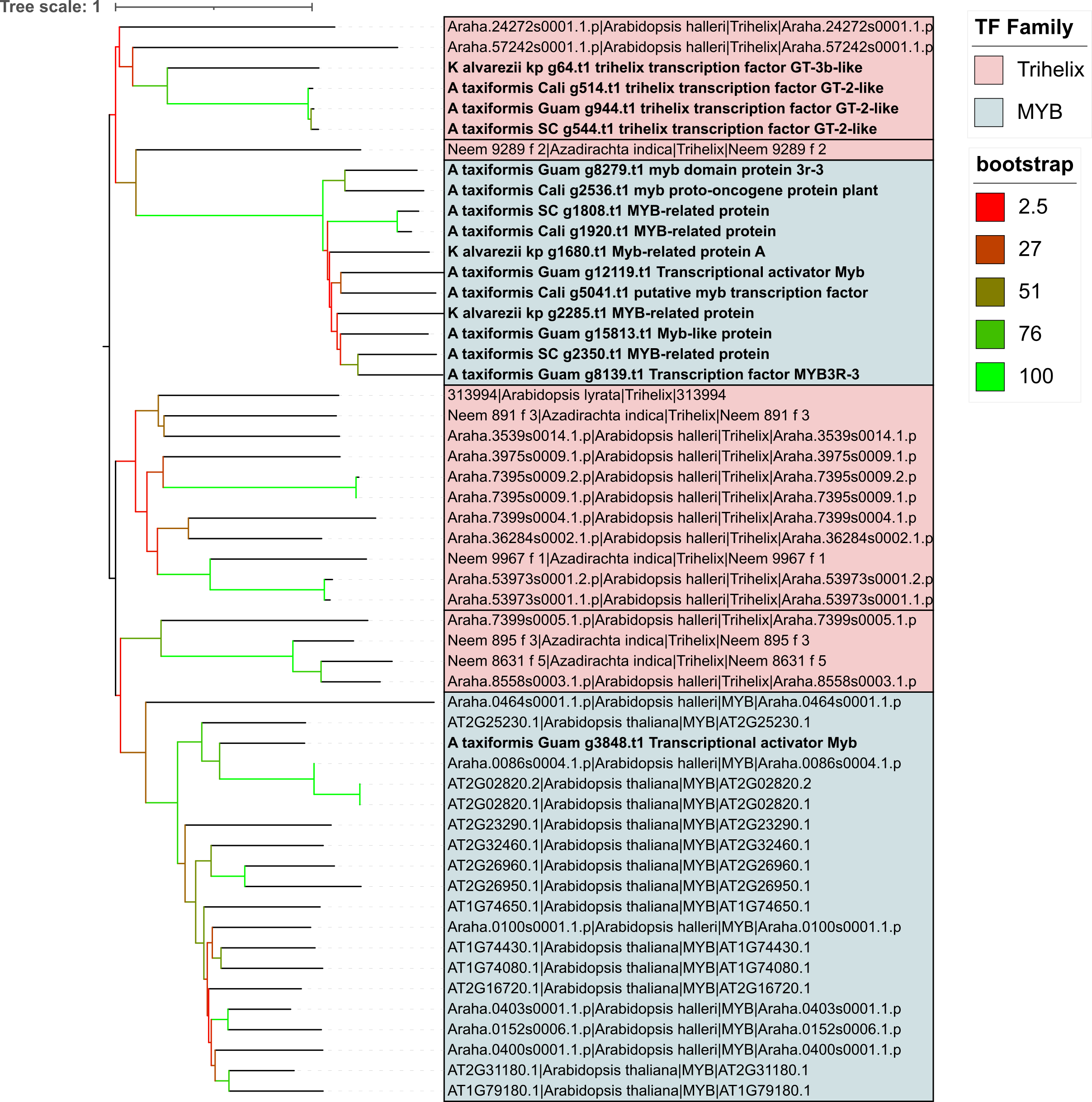

Supplement: Supplementary file 1 [file plants-14-03143-s001.zip › Figure S2.tiff]

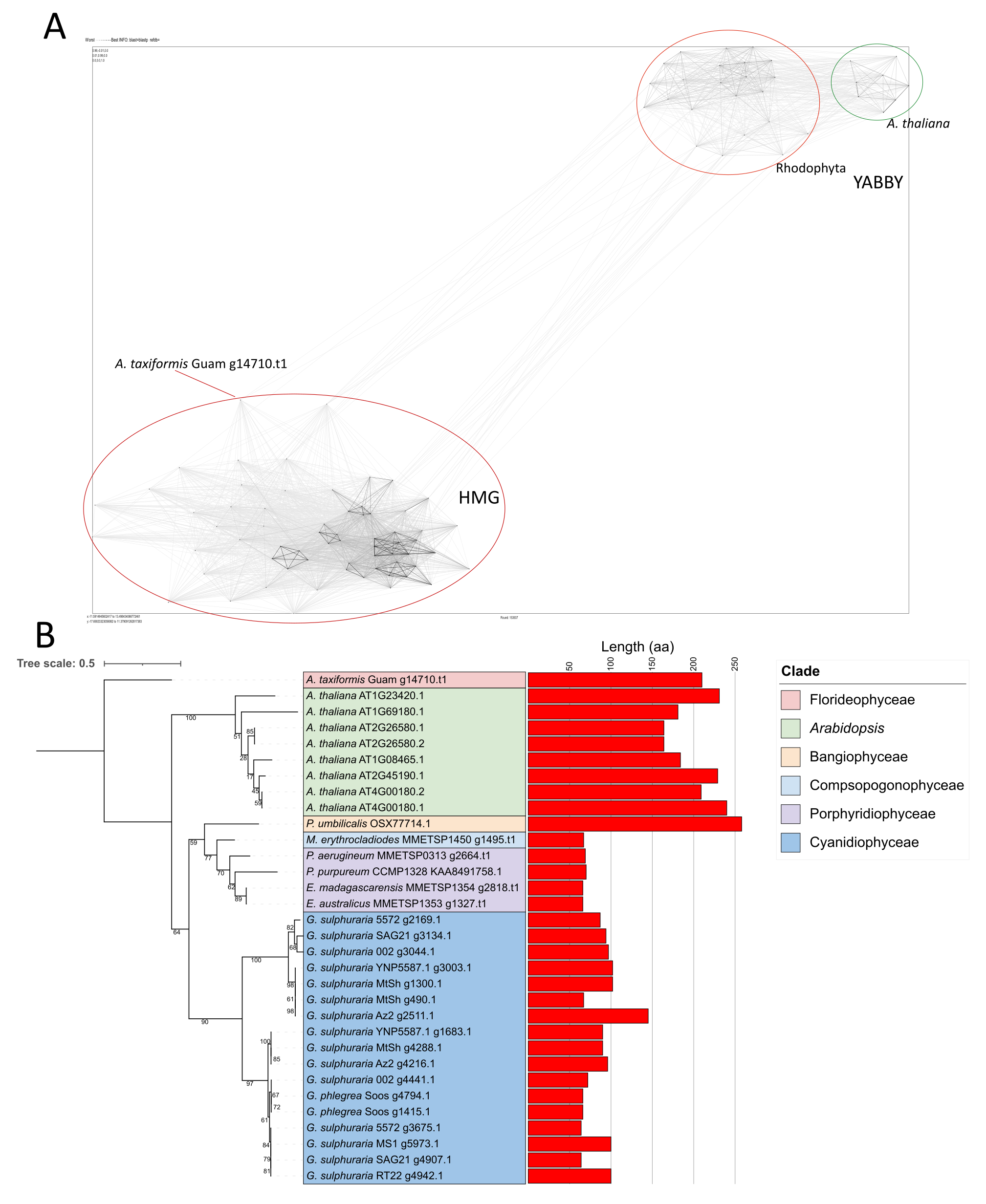

Supplement: Supplementary file 1 [file plants-14-03143-s001.zip › Figure S3.tiff]
